# Influences of Non-Volatile Components on the Aroma of Strong-Aroma Baijiu by Gas Chromatography-Olfactometry and Recombination-Omission Test

**DOI:** 10.3390/foods14142490

**Published:** 2025-07-16

**Authors:** Yingqi Zhou, Yihong Wang, Jia Zheng, Siyi Pan, Xiaoyun Xu, Fang Yuan

**Affiliations:** 1 College of Food Science and Technology, Huazhong Agricultural University, Wuhan 430070, China; zhou2002yq@163.com (Y.Z.); wyihong@webmail.hzau.edu.cn (Y.W.); pansiyi@mail.hzau.edu.cn (S.P.); xuxiaoyun@mail.hzau.edu.cn (X.X.); 2 Wuliangye Yibin Co., Ltd., Yibin 644000, China; zhengwanqi86@163.com; 3 Hubei Key Laboratory of Fruit & Vegetable Processing & Quality Control, Huazhong Agricultural University, Wuhan 430070, China

**Keywords:** strong-aroma baijiu, GC-O, non-volatile compounds, recombination, omission

## Abstract

Aroma is an important indicator for evaluating the quality of baijiu. In this study, we determined the aroma-active compounds in four representative brands of strong-aroma baijiu from Sichuan and Jianghuai regions through GC-MS/O, and GC-TOF-MS quantification. In addition, the non-volatile composition of four baijiu samples was quantified by BSTFA derivatization and GC-MS. By constructing a full recombination model containing both volatile and non-volatile components, the effect of different groups of non-volatile compounds on the aroma of strong-aroma baijiu was evaluated through recombination-omission tests. A total of 72 aroma-active compounds and 59 non-volatile compounds were identified and quantified. The results indicated that pyrazines, furfural, and furan derivatives displayed higher aroma intensities in strong-aroma baijiu produced in Sichuan compared to that produced in Jianghuai. The recombination model that included both aroma-active and non-volatile compounds showed a closer resemblance to the original baijiu samples, underscoring the critical role these compounds play in shaping the dominant aroma profile of strong-aroma baijiu. Non-volatile compounds significantly influenced six aroma attributes: fruity, sweet, sauce, pit, acidic, and alcoholic notes. Omission tests revealed that among posorly volatile organic acids, monobasic acids had distinct effects on the aroma profile, while dibasic acids did not show any noticeable influence on the sensory characteristics.

## 1. Introduction

Baijiu is a traditional Chinese distilled liquor, the production technique of which can be dated back 5000 years. Alongside brandy, whisky, gin, vodka, and rum, baijiu is recognized as one of the six world-renowned distilled spirits [1,2]. Drinking baijiu neat is the most traditional and classic way to enjoy it. This method fully preserves the original aroma and taste of baijiu, allowing drinkers to appreciate its intricate flavor profile. When consuming baijiu neat, it is typically poured into specialized small cups, which usually hold 20 to 30 mL. Baijiu is made from sorghum, wheat and other grains through saccharification, fermentation, distillation, aging and other processes. Due to the diversity of terrain and climate, raw material, manufacturing process and other factors, baijiu can be divided into approximately 12 different aroma types. Among them, soy sauce-aroma, light-aroma, and strong-aroma types are considered as the three basic aroma types due to their widely known flavor characteristics [3]. Soy sauce-aroma baijiu is characterized by its high acidity, presenting a slightly yellow and transparent appearance [4]. It boasts a prominent soy sauce-like aroma, with a delicate taste, long aftertaste and a lingering fragrance that remains in the empty cup. The key flavor compound of light-aroma baijiu is ethyl acetate. Light-aroma baijiu is colorless and transparent, with a pure, sweet, and balanced flavor, and the aftertaste is clean [5]. Strong-aroma baijiu is characterized by a high content of ethyl hexanoate, with short-chain fatty acids and their ester derivatives making a major contribution to its distinctive flavor [6]. Strong-aroma baijiu can be further divided into Sichuan category and Jianghuai category. The main style of the Sichuan category is more intense, with a prominent pit and grain aroma, and the aftertaste is slightly bitter. while the aroma of Jianghuai category is more mellow and moderate, with typical aromas such as floral and fruity, honey, and grassy [7]. Wang et al. [8] reviewed the GC-O and AEDA data from Jianghuai region strong-aroma baijiu (Yanghe Daqu and Gujinggong jiu) and Sichuan region strong-aroma baijiu (Luzhou Laojiao). They found that the Sichuan region strong-aroma baijiu has a higher content of esters and acids, which may be the reason for the difference in flavor between the two types of strong-aroma baijiu.

The typical flavor of strong-aroma baijiu is the result of complex interactions between various aroma compounds as well as non-volatile compounds contained in it. More than 861 trace components have been found in strong-aroma baijiu and 141 trace components have been considered as the main aroma compounds [8]. Non-volatiles, although not directly affecting aroma, were found to be crucial in modulating flavor and overall flavor balance in baijiu [9]. Studies on the flavor contribution of non-volatile compounds to baijiu have mainly focused on taste and mouthfeel. Recent studies have shown that the non-volatiles could enhance the aroma release of most key aroma-active compounds in soy sauce-aroma type baijiu [10], thus affect the aroma. Another study of Laobaigan baijiu reported that non-volatile compounds reduced the alcoholic and sweet odors, while amplifying fruity, acidic, floral, jujube and grain notes [11]. Non-volatile components in baijiu have diverse structures and chemical properties; how these compounds influence the aroma of baijiu remains unclear.

The primary objective of this study was to elucidate the contribution of non-volatile compounds to the aroma profile of strong-aroma baijiu. GC-MS is the primary analytical technique used to classify baijiu. However, the production of baijiu involves a complex series of fermentation, distillation, and aging processes, which leads to the presence of many different compounds [12]. As a result, the limitations in separation and sensitivity of GC-MS may cause potential biomarkers to be overlooked. In this context, GC-TOF-MS offers improved coverage and sensitivity, making it more suitable for the comprehensive analysis of compounds in this complex matrix [13]. In this study, aroma-active compounds in four representative brands of strong-aroma baijiu from Sichuan and Jianghuai regions were systematically identified and quantified using GC-MS/O and GC-TOF-MS techniques. Simultaneously, the non-volatile components in these baijiu samples were accurately determined via BSTFA derivatization coupled with GC-MS analysis. Subsequently, a full recombination model integrating both volatile and non-volatile compounds was constructed. Through recombinationomission tests, the impact of distinct groups of non-volatile compounds on the characteristic aroma of strong-aroma baijiu was evaluated, aiming to provide a deeper understanding of the complex flavor formation mechanism in baijiu.

## 2. Materials and Methods

### 2.1. Baijiu Samples

The baijiu samples were purchased from a local liquor store (Wuhan, China). The details of the samples are shown in Table 1.

### 2.2. Chemicals

All qualitative and quantitative reference standards used were of chromatography grade, with a purity of at least 97%, and were procured from Jizhi Biochemical Technology Co., Ltd. (Shanghai, China). Ethanol, of chromatography grade, was purchased from Aladdin Reagent (Shanghai, China) Co., Ltd. n-Alkanes (C7~C40), 4-octanol, and salicin, all of chromatography grade, were obtained from Sigma-Aldrich (St. Louis, MO, USA). BSTFA (10% TMCS), of chromatography grade, was sourced from TCI (Shanghai, China) Development Co., Ltd. Pyridine and dichloromethane, both with a purity of 99.9%, were acquired from Aladdin Reagent (Shanghai, China) Co., Ltd. Sodium chloride, anhydrous sodium sulfate, and sodium carbonate, of chemically pure grade, were purchased from Sinopharm Chemical Reagent Co., Ltd. (Shanghai, China). Concentrated hydrochloric acid, of analytical reagent grade, was also obtained from Sinopharm Chemical Reagent Co., Ltd. (Shanghai, China).

### 2.3. GC-MS/O Analysis

The GC-MS/O analysis was performed following the modified method described by Niu et al. [14]: 50 mL of the baijiu samples were diluted with Milli-Q water to reach an ethanol concentration of 15%, and 1.5g NaCl was added, then extracted 3 times with CH_2_Cl_2_ (50 mL each time). The combined extract (approximately 150 mL) was extracted three times with Na_2_CO_3_ (50 mL each time, 0.25 mol/L, pH 10.0), and then washed with 50 mL saturated NaCl solution. The neutral/alkaline fraction obtained was marked NBF. The combined aqueous phase (approximately 200 mL) was adjusted to pH 2.0 with HCl (4.0 mol/L) and extracted three times with CH_2_Cl_2_ (70 mL each time) to obtain the acidic fraction named AF. After being dried with anhydrous Na_2_SO_4_, the two fractions were concentrated to 500 μL by using a Wechsler fractionation column and stored at −20 °C before GC-O analysis.

Three trained panelists were selected to conduct the GC-O analysis. During the GC operation, the panelists positioned their noses close to the sniffing port and responded to the intensity of the eluted aroma, recording the aroma descriptors (Odor), intensity values, and corresponding retention times. Intensity was assessed using a six-point scale ranging from 0 to 5: 0 represented “none,” 1 represented “very weak,” 2 represented “weak,” 3 represented “medium,” 4 represented “strong,” and 5 represented “very strong.” The Osme value for aroma intensity was calculated as the average of the ratings provided by the three panelists.

The GC-O instrument was a Trace1300-ISQ7000 (Thermo, Waltham, MA, USA) equipped with a ODP3 sniffing port (Gerstel, Linthicum Heights, MD, USA). A DB-WAX column (30 mm × 0.25 mm × 0.25 μm) was used. The injector temperature was set at 250 °C, and the injection was performed in splitless mode. Helium (purity ≥ 99.999%) was used as the carrier gas in constant linear velocity mode with a flow rate of 1.0 mL·min^−1^. The temperature program was as follows: the initial temperature was set at 40 °C, increased to 50 °C at a rate of 10 °C·min^−1^, held for 10 min, then increased to 80 °C at a rate of 3 °C·min^−1^, held for 10 min, and finally increased to 250 °C at a rate of 5 °C·min^−1^ and held for 5 min. The temperature at the sniffing port was maintained at 250 °C. The EI voltage was set at 70 eV. The ion source temperature was 280 °C, the transfer line temperature was 250 °C. Mass spectra were acquired over a mass range of 35–350 amu. Solvent delay was 3.5 min.

Compounds were identified by comparing the odor, retention indices (RI), mass spectrum, and standard compounds (Std). Volatile compounds were initially identified by searching the NIST 20 library, and Std were subsequently analyzed under the same conditions. Final identification was achieved by comparing the RI and mass spectra of volatile compounds in the samples with those of the Std. RI were calculated based on the retention times of C6–C40 n-alkanes obtained under the same GC-MS analysis conditions.

### 2.4. SPME-GC-TOF-MS Analysis

Each sample was diluted with Milli-Q water to achieve a final ethanol content of 5%. In a 20 mL headspace vial, 5 mL of the diluted baijiu sample and 2 g of saturated NaCl were added, followed by the addition of 5 μL of 4-octanol (20,860 mg/L). The vial was sealed. The sample was allowed to equilibrate at 45 °C for 15 min, after which an SPME fiber (2 cm, 50/30 μm DVB/CAR/PDMS) was used for extraction. The extraction time was 30 min. The SPME fiber was desorbed in the GC injector port maintained at 250 °C for 5 min in splitless mode. The SPME-GC-TOF-MS analysis was performed following the modified method described by Yu et al. [12]. The chromatographic column used was a DB-WAX column (30 mm × 0.25 mm × 0.25 μm). Helium (purity ≥ 99.999%) was used as the carrier gas in constant flow mode at a flow rate of 1.0 mL·min^−1^. The temperature program was as follows: the initial temperature was set at 40 °C, held for 5 min, then increased to 220 °C at a rate of 5 °C·min^−1^, followed by an increase to 250 °C at a rate of 20 °C·min^−1^ and held for 7.5 min. The ion source emission current was set as 1 mA. The voltage of the EI source was set at 70 eV, the ion source temperature was 230 °C, and the transfer line temperature was 290 °C. Mass spectra were acquired over a mass range of 35–350 amu at an acquisition rate of 10 spectra/s.

### 2.5. BSTFA Derivatization and GC-MS for Non-Volatile Compound Analysis

A 10 mL sample of baijiu containing 1 mg/L salicin was pre-concentrated to approximately 1 mL using a rotary evaporator, transferred into a 2 mL injection vial, and then completely evaporated under a stream of high-purity nitrogen gas. Subsequently, 100 μL of BSTFA (containing 10% TMCS) and 50 μL of pyridine were added to the prepared sample. The derivatized sample was incubated at 75 °C for 3 h, then 1 μL of the sample was injected into a GC-MS system. The GC Column was an HP-5MS (30 m × 0.25 mm × 0.25 μm). Inlet temperature was 250 °C. Helium (purity ≥ 99.999%) was used as the carrier gas with a flow rate of 1.0 mL·min^−1^. The temperature program was as follows: the initial temperature was set at 50 °C, held for 2 min, increased at 4 °C·min^−1^ to 160 °C and held for 5 min, then increased at 4 °C·min^−1^ to 230 °C and held for 5 min. Split ratio was set at 1:1. EI voltage was set at 70 eV. The ion source temperature was 250 °C, and the transfer line temperature was 280 °C. Mass spectra were acquired over a mass range of 40–400 amu. Solvent delay was 5 min.

### 2.6. Quantitation of Volatile and Non-Volatile Compounds

The compounds were quantified using calibration curves. Standard solutions were prepared by dissolving the reference standards in ethanol and then diluting them with an aqueous ethanol solution (52% vol, adjusted to pH 3.5 with lactic acid) to various gradient concentrations. The pre-treatment and analytical procedures for the standard mixtures at different gradient concentrations were identical to those used for the samples. Calibration curves were plotted with the response ratios of the target compounds to the internal standard (IS) as the ordinate and the concentration ratios as the abscissa. The fitness of the calibration curves was evaluated using the coefficient of determination (R^2^). The limits of detection and quantification were defined as the lowest concentrations on the calibration curves corresponding to signal-to-noise ratios of 3 and 10. For compounds without available reference standards, semi-quantification was performed relative to the concentration of the IS. A known quantity of authentic standards was added to a mixed baijiu sample to determine the recovery rate. The calculation of recovery rate is as follows: Recovery rate (R) = (Measured value of spiked sample − Background value of original sample)/Theoretical amount of spike × 100%.

### 2.7. Odor Active Values (OAVs) of Volatile Compounds

The OAV of volatiles in baijiu was derived by comparing their concentrations to their thresholds in ethanol solution, and in general, compounds with an OAV ≥ 1 are considered significant contributors to the overall aroma profile. The odor thresholds used in this study are derived from previous research [1,7,15,16].

### 2.8. Aroma Recombination Test

Two recombination models were established: Rec A (consisting of 70 quantified volatiles identified in GC-O) and Rec B (consisting of 70 quantified volatiles identified in GC-O and 54 quantified non-volatile compounds). The compounds were added to an aqueous ethanol solution (52% vol) according to their quantitative results. The samples were then allowed to stand and reach equilibrium, enabling further evaluation and analysis of the baijiu samples and their corresponding recombination models.

### 2.9. Aroma Omission Test

The recombination models were recombined using the triangle test method, assigned random three-digit codes, and their order was randomized. Panelists were required to identify, from the three samples presented, the one that exhibited sensory differences compared to the other two samples, describe the observed differences, and score the difference (0 = identical, 1 = slightly different, 2 = considerably different, 3 = completely different). Each experiment was repeated twice.

### 2.10. Quantitative Descriptive Analysis (QDA)

A sensory evaluation panel comprising ten trained panelists, consisting of five males and five females, was selected based on their olfactory acuity and odor description capabilities. The study was approved by the HZAU Institutional Review Board (IRB).

Prior to the formal QDA, training in basic aroma detection and scale usage was conducted for the panelists. During the training period (six sessions, each lasting one hour), various representative samples of strong-aroma baijiu were provided to the panelists. Discussions and screening of odor were engaged in by the panelists with reference to the sensory descriptors in the baijiu flavor wheel. Finally, 10 aroma descriptors (alcoholic, roasted, pit aroma, flower, fruity, grain, acidity, sweet, grass, sauce) were selected to characterize the flavor characteristics of strong-aroma baijiu. The group also discussed the definition of the best descriptor and unified the evaluation criteria for aroma descriptors (Table 2). Overlapping concepts were eliminated, and it was ensured that the descriptors could comprehensively cover all samples. The panelists were also trained to correctly use the selected terminology. In the formal experiment, the samples were evaluated using a five-point scale, where 1 denoted “weak” and 5 denoted “strong.” Baijiu samples of 10 mL were placed in tasting glasses labeled with three-digit random numbers and covered with lids to prevent sample volatilization. The samples were presented in a randomized order. All sensory experiments were conducted in the sensory laboratory of Huazhong Agricultural University, which was designed in accordance with the ISO 8589 standard [17]. The room temperature was kept at 22 °C. The baijiu samples were allowed to reach room temperature before the test. The panelists were seated in individual booths and were required to take a break of at least 5 min between evaluating each set of samples.

### 2.11. Data Analysis

Qualitative analysis of substances was conducted using Qualitative analysis 10.0 software and matched with the NIST 20 library. Statistical analyses were conducted using SPSS Statistic 22.0 software. One-way analysis of variance (ANOVA) was performed on the compound concentrations, and results were considered statistically significant if *p* < 0.05. Principal component analysis (PCA) was carried out using an online platform (https://www.metaboanalyst.ca/). Data visualization was performed using Origin 2022 and an online graphing tool (https://www.chiplot.online/).

## 3. Results

### 3.1. Aroma-Active Compounds in Strong-Aroma Baijiu

For the purpose of facilitating GC-O/MS analysis and compound identification, the samples were further separated into AF and NBF fractions to reduce complexity. Following this procedure, a total of 72 aroma compounds were identified through the combination of liquid–liquid extraction and GC-O analysis, including 34 esters, nine alcohols, nine acids, nine aldehydes, four ketones, two furans, two aromatic compounds, two pyrazines, and one sulfide (Table 3). The results showed that acids, alcohols and high content esters were more abundant in AF, which mainly provide cheesy, sweaty, alcoholic and fruity aromas. Aldehydes and heterocyclic compounds were mainly detected in NBF, which provide pungent, fruity and roasted odors.

Among the 34 aroma-active esters, ethyl hexanoate, as the dominant ester compound characteristic of strong-aroma baijiu, consistently exhibited an odor intensity exceeding 4 across all four samples. This compound imparts a strong fruity note to the baijiu. Furthermore, several other esters were present at relatively high concentrations, such as ethyl acetate, ethyl butyrate, isoamyl acetate, ethyl valerate, isoamyl butyrate, and ethyl octanoate (each with an odor intensity ≥3).

A total of nine acid compounds were identified in this study. Their odor descriptors were predominantly characterized as acidic or sweaty. Among these, acetic acid and butyric acid exhibited odor intensities greater than 3.

Of the nine aroma-active alcohols, isoamyl alcohol demonstrated the highest odor intensity, reaching 3.8, indicating its highest odor contribution. Furthermore, n-propanol, isobutanol, n-butanol, and n-hexanol consistently displayed odor intensities exceeding 2 across nearly all four samples.

Among the seven aroma-active aldehydes, isobutyraldehyde and phenylacetaldehyde (floral note) both exhibited odor intensities greater than 2. Analysis of odor descriptors revealed that aldehydes predominantly possess pungent aroma characteristics, frequently described as grassy or green.

Beyond the aforementioned esters, acids, alcohols, and aldehydes, other chemical classes also make significant contributions to the aroma profile of strong-aroma baijiu, including ketones, phenols, furans, and pyrazines. Specifically, 2,3-butanedione (odor intensity > 1) and acetoin (odor intensity > 3) impart sweet, buttery notes. 4-Methylphenol (odor intensity > 1.8) contributes smoky aromas. Furfural (odor intensity > 1.8) and 5-methylfurfural (odor intensity > 1.5) provide caramel-like and baked notes. 2-Acetylfuran (odor intensity > 1.5) exhibits almond and nutty characteristics. Dimethyl trisulfide (odor intensity > 2) contributes a sulfurous off-note.

### 3.2. Quantification of Aroma-Active Compounds by SPME-GC-TOF-MS

Among the 72 aroma-active compounds in four samples, 70 compounds were successfully quantified using SPME-GC-TOF-MS with authentic standards (Table 4 and Table S1). 2,6-Diethyl pyrazine and tetramethyl pyrazine, although tentatively identified in GC-O, could not be detected by SPME-GC-TOF-MS due to their extremely low concentration, thus were not included in the further study. We also found that the repeatability of a few compounds in the sample matrix was worse than that in the synthetic matrix. This may be due to the complex composition of baijiu, which affects the adsorption stability of SPME fiber.

OAVs were also calculated according to the quantification data (Table 4). Excepting the compounds without published odor thresholds, a total of 49 aroma compounds with OAV > 1 were obtained. In the strong-aroma baijiu samples, the OAV of ethyl hexanoate was the highest, followed by ethyl butyrate, ethyl valerate and ethyl caprylate. 1-Butanol and its OAV were in the forefront among alcohols, followed by 1-hexanol. The OAV of butyric acid and hexanoic acid were significantly higher than those of other acids. Furfural was the aldehyde with highest concentration and OAV. Due to the low threshold [18], the OAV of 2-pentanone was significantly higher than that of other ketones.

**Figure 1:**
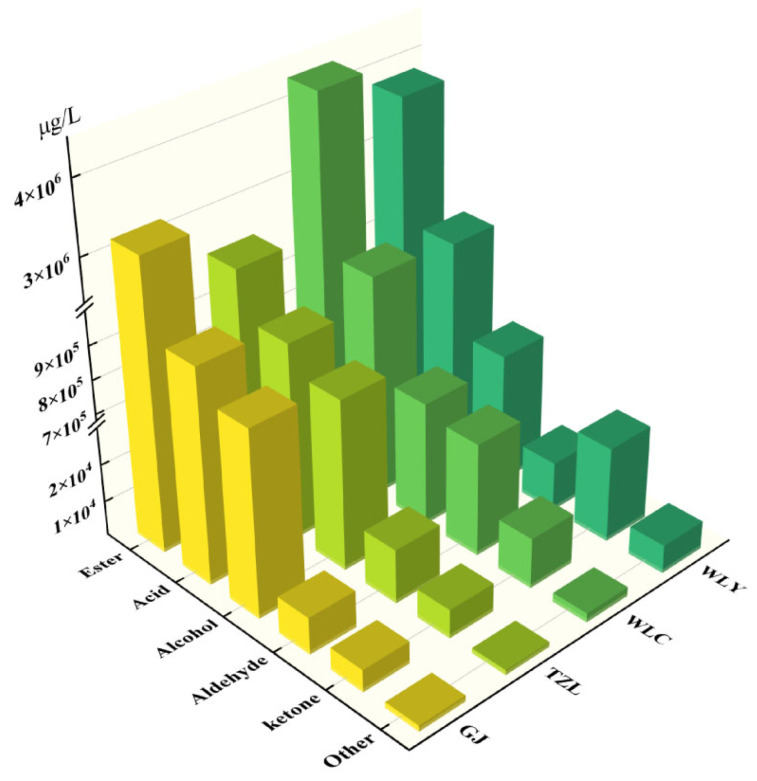
Total concentrations of volatile compounds by category in strong-aroma baijiu samples.

Comparing the volatile compounds in strong-aroma baijiu samples from different regions (Figure 1), it could be found that samples from the Sichuan region exhibited generally higher total concentration of esters, while those from the Jianghuai region contained more alcohols compared to Sichuan samples. In contrast, concentrations of other compound categories showed no significant geographical variations and were primarily brand-dependent.

### 3.3. Qualitative and Quantitative Analysis of Non-Volatile Compounds

**Figure 2:**
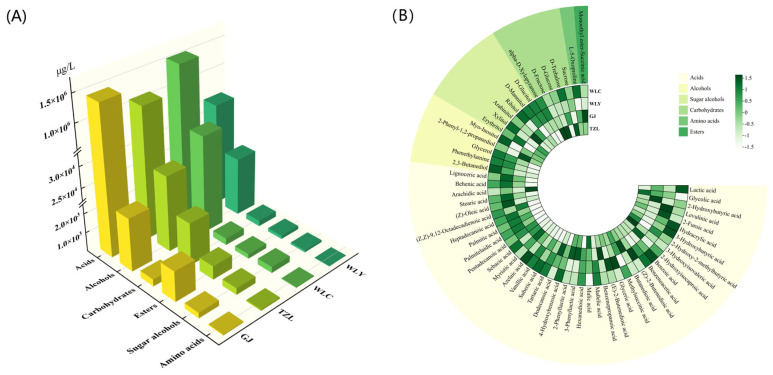
Concentration of non-volatile compounds in strong-aroma baijiu. (**A**) Total concentration by category; (**B**) heatmap of non-volatile compounds.

A total of 59 non-volatile compounds were identified by combining BSTFA derivatization with GC-MS technology, including 41 acids, five alcohols, six sugar alcohols, five carbohydrates, one amino acid, and one ester (Figure 2). Out of them, 55 non-volatiles were quantified using authentic standards. Meanwhile, hydracrylic acid, glyceric acid, lignoceric acid, and 2-phenyl-1,2-propanediol were semi-quantified due to the absence of available standards. The quantification data can be found in Table S2. Among the non-volatile compounds in strong-aroma baijiu samples, organic acids predominated, followed by alcohols, while amino acids, sugar alcohols, and sugars were present at comparatively lower levels.

In the four strong-aroma baijiu samples, lactic acid, palmitic acid and (Z)-oleic acid were the most abundant non-volatile organic acids. Among them, lactic acid reached the highest level of 1508.54 mg/L in the WLC sample, far more than other organic acids. In addition, the concentration of 2-hydroxyisocaproic acid, (Z,Z)-9,12-octadecadienoic acid was also high. Glycerol and 2-phenyl-1,2-propanediol were the most abundant non-volatile alcohols in strong-aroma baijiu. In addition, only one amino acid, L-5-Oxoproline, was detected in strong-aroma baijiu. The non-volatile profile of strong-aroma baijiu was brand-dependent. No obvious trends were observed between the two regions.

### 3.4. Aroma Recombination

To construct the aroma recombination model, we prepared a reconstituted sample (Rec A) by precisely blending 70 quantified volatile compounds based on their individual quantitative results. An additional 55 non-volatile compounds were incorporated into Rec A to form the full recombination sample (Rec B). Rec A, Rec B and the original baijiu samples were subjected to QDA analysis.

**Figure 3:**
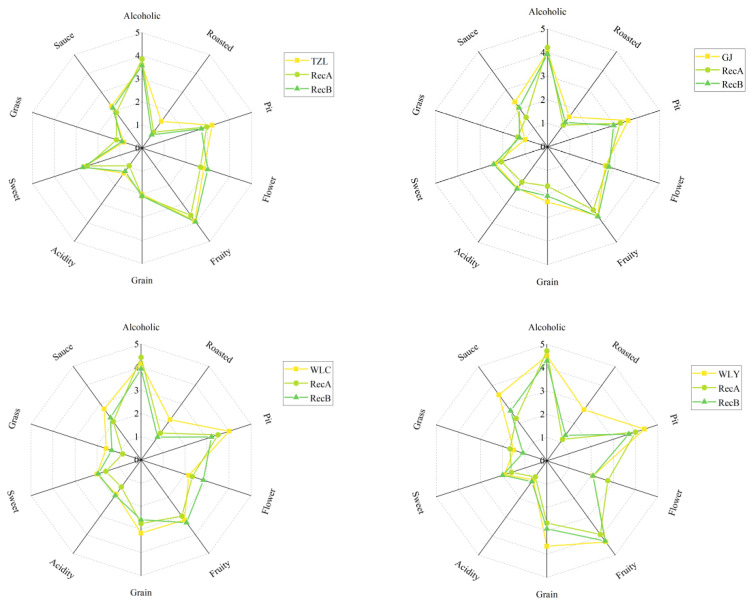
Scores of aroma attributes of the recombination models Rec A and Rec B and baijiu samples.

During the QDA training period, the reviewers finally selected 10 aroma descriptors (alcoholic, roasted, pit, fruity, flower, grain, acidity, sweet, grass, and sauce) to characterize the aroma characteristics of strong-aroma baijiu samples. The results of QDA (Figure 3) showed that the aroma profile of the recombination samples was similar to the original baijiu samples, indicating that the aroma-active compounds selected in this study were qualified and quantified reliably. Compared with Rec A, the aroma profile of Rec B (added non-volatile compounds) were closer to the original baijiu samples, specifically in the aspects of sweet, fruity, acidity and grass notes. However, there were still differences in roasted and sauce aroma between the reconstructed samples and the original samples.

**Figure 4:**
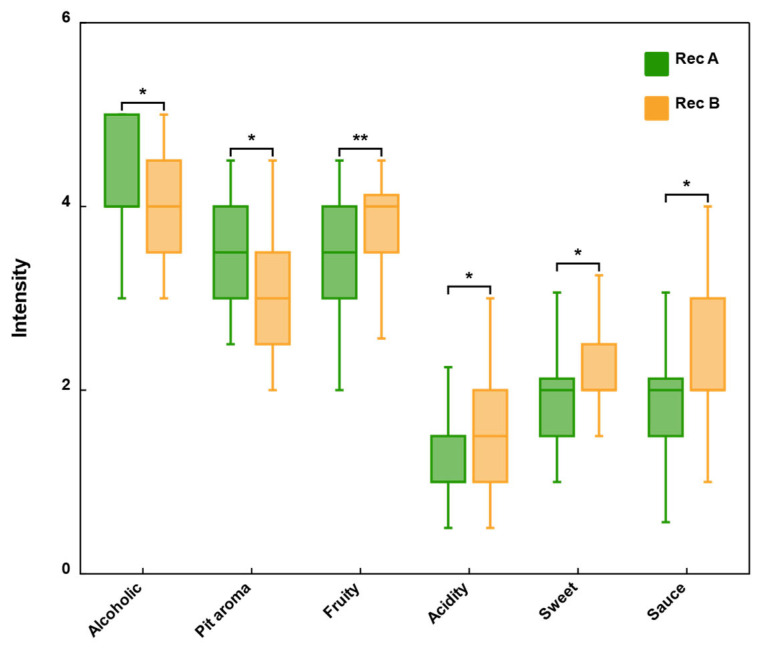
Six aroma attributes with significant differences between Rec A and Rec B. *, significant when *p* < 0.05; **, significant when *p* < 0.01.

T test was used to determine the difference between the average score of each descriptor in Rec A and Rec B, so as to further explore the effect of non-volatile compounds on the aroma characteristics of baijiu. As shown in Figure 4, six descriptors (fruity, sweet, sauce, acidity, alcoholic and pit aroma) had significant differences between Rec A and Rec B (*p* < 0.05), while there was no significant difference in flower, roasted, grass and grain aroma. The aroma intensity of fruity, sweet, sauce and acidity in Rec B was significantly higher than that in Rec A (*p* < 0.01), especially the fruity aroma, while alcoholic and pit aroma were stronger in Rec A.

### 3.5. Aroma Omission Test

To determine the contribution of non-volatile compounds to the overall aroma of strong-aroma baijiu, we conducted an omission test. The non-volatile compounds were divided into four groups according to their structures (Table 5): organic acids (OM1), sugars (OM2), sugar alcohols (OM3) and alcohols (OM4). One group of compounds was omitted from the full recombination model (Rec B) each time. No difference was observed by triangle test when OM2, OM3 and OM4 groups were omitted from the full model (*p* > 0.05). When OM1 was absent, the overall aroma profile showed a perceivable difference compared with the full model (*p* < 0.001). Specifically, the intensity of acidity and fruity decreased, while the perception intensity of alcoholic aroma increased.

In order to further analyze the contribution of organic acids to the aroma of strong-aroma baijiu, non-volatile organic acids were further divided into monobasic acid (OM5) and dibasic acid (OM6) for the aroma omission test. The sensory result showed that compared with the full model, the intensity of acidity and fruity notes was significantly reduced in the OM5 sample, but the alcoholic aroma was unchanged. There was no sensory difference between OM6 and the full model (*p* > 0.05).

## 4. Discussion

Strong-aroma baijiu possesses a highly complex aroma profile. In this study, the results from GC-O and quantification revealed that several compounds, including ethyl butyrate, ethyl pentanoate, ethyl hexanoate, butyl hexanoate, ethyl 3-methylbutyrate, and hexanoic acid, significantly contribute to the aroma characteristics of strong-aroma baijiu. These findings are consistent with reports in previous studies that esters, especially ethyl ester compounds, were the most important aroma compounds in baijiu [19,20,21]. The aroma composition of baijiu warrants further investigation because, in addition to the essential role of the main compounds in defining its overall aroma profile, certain trace components with low odor thresholds also significantly influence its aromatic characteristics, such as furan derivatives, sulfur-containing compounds, and pyrazines.

It is important to note that both the Osme values and the concentrations of most aroma compounds varies significantly across different samples. Generally, samples from the Sichuan region exhibited generally higher total concentration of esters (e.g., ethyl pentanoate, amyl acetate, methyl hexanoate), while those from the Jianghuai region contained more alcohols compared to Sichuan samples. Other variations in trace components also likely contribute to the flavor differences in strong-aroma baijiu from various production regions. For instance, compounds such as 2-heptanone, 2,6-diethylpyrazine, tetramethylpyrazine, 2-acetylfuran, and 5-methylfurfural showed higher aroma intensities in samples WLC and WLY from the Sichuan region compared to the YH and GJ samples from the Jianghuai region. In particular, heterocyclic compounds contribute to roasted, baked, and smoky aroma characteristics. Previous studies have highlighted that 2,6-dimethylpyrazine is a crucial aroma compound in strong-aroma baijiu [22], while tetramethylpyrazine offers a unique flavor reminiscent of popcorn and nuts [23], which were also the key aroma-active compounds in the fermentation starter (Daqu) of strong-aroma baijiu [24]. Additionally, furfural has been identified as an important compound for bitterness and baked notes in baijiu [25]. Furthermore, 2-acetylfuran has been found in strong-aroma baijiu, contributing to its roasted aroma [21]. Thus, it can be inferred that heterocyclic compounds such as furans and pyrazines are characteristic aroma compounds that distinguish strong-aroma baijiu from those produced in the Jianghuai and Sichuan regions, consistent with the findings reported by He et al. [26]. We observed that compounds such as isobutyl acetate, ethyl octanoate, and ethyl isovalerate were present in higher concentrations in the YH and GJ samples compared to the WLC and WLY samples. This difference might be attributed to the lower fermentation temperature (usually between 25–32 °C) of the Jianghuai samples, which likely allowed for the retention of more short-chain esters [27].

In the recombination tests, aroma-active compounds were found to be crucial for the aroma of baijiu, while non-volatile compounds also significantly influenced its aroma profile. The scores for most aroma attributes in Rec B (full model) were significantly higher than those in Rec A (only volatiles). This indicates that non-volatile compounds have a notable enhancing effect on aroma perception. This enhancement may be due to non-volatile compounds altering the liquid–air partition rate of the volatiles, thereby increasing the amount of volatiles in the headspace. Our results are aligned with a recent report on soy sauce-aroma baijiu that the intensities of floral, fruity, acidic, and grain aromas of the fourteen key aroma-active compounds were enhanced in the presence of non-volatiles [10]. Wang et al. [16] specifically studied the perceptual interactions between lactic acid and ethyl lactate and found that lactic acid created additive or synergistic odor effects with both ethyl lactate and ethyl acetate.

However, in this study, not all aromas were enhanced by the non-volatile compounds. In fact, the intensity of the pit and alcoholic aromas was more pronounced in Sample Rec A than in the original. This indicates that non-volatile compounds may mask these two specific aromas in baijiu. It is also possible that the heightened fruity notes suppressed the perception of the most concentrated compounds, which contribute to the pit aroma (mainly from ethyl hexanoate) and the alcoholic aroma (mainly from ethanol).

The results of the omission test confirmed the contribution of non-volatile acids to the aroma of baijiu. The results indicated that among the non-volatile organic acids, monobasic acids primarily contributed to the aroma profile of baijiu, providing more sour and fruity notes. In this study, lactic acid is the most abundant non-volatile acid in strong-aroma baijiu, directly contributing to the acidic taste. Therefore, a deficiency in monobasic acids would directly result in a decrease in the overall acidity of the baijiu [28]. It should be noted that some of the monobasic acids were semi-volatile, especially at higher temperatures, so it is not surprising that monobasic acids could enhance the sour aroma of baijiu. The enhancement of fruity aroma can likely be attributed to the change in gas–liquid partitioning of esters in the presence of monobasic acids [29]. Meanwhile, organic acids are important precursors for esterification to produce esters, and esters are the key compounds to shape the flavor of baijiu. They provide aroma characteristics such as fruit and flower. For instance, ethyl laurate is believed to impart fruit and flower aroma to baijiu, making a substantial contribution to its flavor [30,31]. Dodecanoic acid, palmitic acid, and oleic acid have also been confirmed as precursors to some fatty acid esters [32]. Additionally, the enhancement of fruity notes might also result from the synergistic effect between the sour aroma and the fruity aroma.

During the omission test, we also observed that omitting both monobasic and dibasic acids (OM1) allowed more panelists to perceive the difference compared to omitting only monobasic acids (OM5). Although not statistically significant, some panelists perceived an increase in alcoholic aroma in OM1 compared to OM5, indicating that the dibasic acids could mask the alcoholic notes but had little effect on other aroma characteristics. Liu et al. [33] discovered that long-chain fatty acids, such as oleic acid, influenced the interactions between volatile compounds and ethanol/water molecules in baijiu. Specifically, these long-chain fatty acids inhibited the volatilization of ethanol, thereby helping to reduce the pungent odor of baijiu. Zhang et al. found that organic acids can inhibit the volatility of n-butanol due to intermolecular van der Waals forces and electrostatic interactions [34]. These findings align with the results of the current study.

## 5. Conclusions

In this study, a total of 72 volatile compounds and 59 non-volatile compounds were identified and quantified using GC-TOF-MS and GC-MS, respectively. Recombination experiments successfully replicated the characteristic aroma profile of strong-aroma baijiu, validating the comprehensive contribution of these compounds. Aroma omission tests revealed that the absence of non-volatile organic acids significantly diminished sour and fruity notes while slightly intensifying alcoholic pungency. Further subdivision of organic acids into monobasic and dibasic categories indicated that monobasic acids primarily enhanced the acidic and fruity aromas, whereas dibasic acids slightly mitigate the alcoholic aroma, suggesting their role in harmonizing and masking undesirable sensory attributes.

This research not only comprehensively analyzed the volatile and non-volatile components of strong-aroma baijiu but also elucidated their critical roles in flavor formation. These findings provide a scientific foundation for quality control and product innovation in the baijiu industry, particularly regarding the role of non-volatile compounds in modulating aroma complexity and balance. However, this study only discusses the odor, and the mechanism of the impact of these non-volatile compounds on the aroma of baijiu needs further research.

## Figures and Tables

**Table 1:** Information about the baijiu samples.

No.	Brand	Label	Region	Alcohol Content (% vol)	Manufacturer	Location
1	Wuliangchun	WLC	Sichuan	52%	Sichuan Yibin Wuliangye Group Co., Ltd.	Yibin, China
2	Wuliangye	WLY	Sichuan	52%		
3	Gujinggong	GJ	Jianghuai	52%	Anhui Gujing Gongjiu Co., Ltd.	Bozhou, China
4	Tianzhilan	TZL	Jianghuai	52%	Jiangsu Yanghe Distillery Co., Ltd.	Suqian, China

**Table 2:** Definition and reference of the aroma descriptors.

Descriptor	Definition	Reference
Alcoholic	ethanol	15 mL of 52% ethanol aqueous solution
Roasted	baking and nuts	A 52% ethanol aqueous solution of 2,3,5-trimethylpyrazine (5 mg/L)
Pit aroma	ethyl caproate (fruity, waxy, cucumber)	52% ethanol aqueous solution of ethyl hexanoate (100 mg/L) and hexanoic acid (20 mg/L)
Flower	flowers	52% ethanol aqueous solution of phenylethanol (10 mg/L)
Fruity	fruits such as pineapple and banana	52% ethanol aqueous solution of ethyl caproate (500 mg/L) and ethyl butyrate (50 mg/L)
Grain	steamed food from the fermentation and steaming of grains	5 g steamed sorghum
Acidity	fermented grains and saccharification and fermentation agents	52% ethanol aqueous solution of acetic acid (50 mg/L)
Sweet	honey and sweet fruits	52% ethanol aqueous solution of γ-nonanolactone (100 ug/L)
Grass	green grass	52% ethanol aqueous solution of 3-methylbutanal (20 mg/L) and hexanal (20 mg/L)
Sauce	soy sauce	5 mL soy sauce

**Table 3:** The aroma compounds detected in representative strong-aroma baijiu by GC-O.

No.	Aroma Compounds	Odor Description	Fraction	RI (DB-WAX)	LRI	Intensity				Identification Methods
						GJ	TZL	WLC	WLY	
1	Acetaldehyde	Pungent	NBF	714	728	2.5	2.3	1.3	1	RI, Aroma, MS, Std
2	Isobutyraldehyde	Pungent, Malt, Green	NBF	821	826	2.7	2.5	2.7	2	RI, Aroma, MS, Std
3	Ethyl acetate	Pineapple	NBF/AF	888	890	2.5	3.2	3.5	3	RI, Aroma, MS, Std
4	Ethyl propionate	Fruity	NBF/AF	951	943	2	2	1.5	2.5	RI, Aroma, MS, Std
5	Ethyl isobutyrate	Fruity, Sweet, Rubber	NBF	955	948	2	1.7	1.7	1.5	RI, Aroma, MS, Std
6	2,3-Butanedione	Butter	AF	975	981	1.5	1.5	1	3	RI, Aroma, MS, Std
7	Isobutyl acetate	Fruity, Apple, Banana	AF	1018	1015	2	2	1.5	1.7	RI, Aroma, MS, Std
8	2-Pentanone	Gasoline, Rubber	NBF/AF	1020	1000	2.5	2.3	2.5	2.7	RI, Aroma, MS, Std
9	2-Butanol	Wine, Fruity	NBF	1024	1022	1	1.3	1.3	1.3	RI, Aroma, MS, Std
10	Ethyl butyrate	Apple	NBF/AF	1035	1028	3.3	3.2	3.7	3.3	RI, Aroma, MS, Std
11	1-Propanol	Alcohol, Fruity, Banana	AF	1036	1037	3	2.7	2.8	3.2	RI, Aroma, MS, Std
12	Ethyl 2-methylbutyrate	Apple	NBF	1050	1053	2.8	1.7	2.1	1.5	RI, Aroma, MS, Std
13	Ethyl isovalerate	Fruity	NBF/AF	1068	1070	2	2	1	1.5	RI, Aroma, MS, Std
14	Hexanal	Grass	NBF	1078	1083	-	1	1	1.5	RI, Aroma, MS, Std
15	2-Methyl-1-propanol	Bitter	NBF/AF	1092	1087	2.5	2.7	2	2.3	RI, Aroma, MS, Std
16	2-Pentanol	Green, Apple	NBF	1118	1116	1	2	1	1.8	RI, Aroma, MS, Std
17	Isoamyl acetate	Banana	NBF	1124	1125	2.8	2.5	2	2.5	RI, Aroma, MS, Std
18	Ethyl valerate	Fruity	NBF	1134	1135	3	3.3	3.5	3.7	RI, Aroma, MS, Std
19	1-Butanol	Fruity, Sweet	NBF/AF	1142	1141	2.3	2.7	2.5	1	RI, Aroma, MS, Std
20	Amyl acetate	Fruity, Banana	NBF	1175	1178	-	-	1	1	RI, Aroma, MS, Std
21	Methyl hexanoate	Fruity, Fragrance	NBF	1184	1184	1.5	1.8	2.3	2	RI, Aroma, MS, Std
22	2-Heptanone	Pungent	NBF	1182	1190	-	-	1	1	RI, Aroma, MS, Std
23	Ethyl 4-methylpentanoate	Fruity	NBF	1190	1192	-	1.5	1.5	2.5	RI, Aroma, MS
24	3-Methyl-1-butanol	Pungent	NBF/AF	1209	1202	3.8	3.5	3.8	2.8	RI, Aroma, MS, Std
25	Ethyl Hexanoate	Apple, Fruity	NBF/AF	1272	1247	4	4.5	4.5	4.8	RI, Aroma, MS, Std
26	Isoamyl butyrate	Fruity	NBF	1259	1257	2.5	3	2	3	RI, Aroma, MS, Std
27	Acetoin	Butter, Cream	AF	1287	1273	3.3	3	3	3	RI, Aroma, MS, Std
28	Ethyl furfuryl ether	Ink, Gasoline, Paint	NBF	1291	1287	2	1	2.2	2.5	RI, Aroma, MS, Std
29	2-Heptanol	Mushroom	NBF	1322	1328	1.5	1.8	1	2	RI, Aroma, MS, Std
30	Caproic acid propyl ester	Fruity	NBF	1316	1328	-	1.3	-	2.3	RI, Aroma, MS, Std
31	Ethyl heptanoate	Fruity	NBF	1331	1362	1	2	1	2	RI, Aroma, MS, Std
32	Ethyl lactate	Ethanol, Fruity, Sweet	NBF	1347	1350	1.7	1.7	2	1.5	RI, Aroma, MS, Std
33	1-Hexanol	Flower, Green, Grain	NBF/AF	1355	1361	3	1.7	3	3	RI, Aroma, MS, Std
34	Dimethyl trisulfide	Sulfide	NBF	1377	1381	3	3.3	2	3.2	RI, Aroma, MS, Std
35	Butyl hexanoate	Fruity	NBF	1407	1408	1.8	2.7	1.7	2.7	RI, Aroma, MS, Std
36	(E)-2-Octenal	Fat	NBF	1430	1429	-	1	1	1.2	RI, Aroma, MS, Std
37	Ethyl caprylate	Fruity, Fat	NBF/AF	1435	1435	3.3	3.7	3	3	RI, Aroma, MS, Std
38	2,6-diethylpyrazine	Grass, Land, Raw Potatoes	NBF	1444	1442	-	2.7	3	3.5	RI, Aroma
39	Acetic acid	Sour	AF	1449	1448	3.5	3.7	3.8	3.5	RI, Aroma, MS, Std
40	Isopentyl hexanoate	Fruity	NBF	1451	1459	2	3	1.5	2.5	RI, Aroma, MS, Std
41	Furfural	Bread, Almonds, Sweet	NBF/AF	1462	1461	1.8	2	2.3	2	RI, Aroma, MS, Std
42	Tetramethylpyrazine	Bitter, Bakery, Nutty	NBF	1469	1478	-	-	2.8	2.3	RI, Aroma
43	2-Acetylfuran	Balm	NBF	1490	1497	-	1.5	2	2	RI, Aroma, MS, Std
44	Pentyl hexanoate	Fruity	NBF	1501	1507	-	2	1	2.5	RI, Aroma, MS, Std
45	Benzaldehyde	Bitter almond	NBF	1520	1521	-	1.5	1.5	1	RI, Aroma, MS, Std
46	(E)-2-Nonenal	Cucumber	NBF	1534	1535	2	2	1	1	RI, Aroma, MS, Std
47	Furfuryl acetate	Fruity	NBF	1539	1541	1	1.5	1.5	1	RI, Aroma, MS, Std
48	Isobutyric acid	Rancidity, Cheese	AF	1563	1559	2	2	1	1.5	RI, Aroma, MS, Std
49	5-Methyl furfural	Caramel	NBF	1570	1565	1.5	2	2.5	2.5	RI, Aroma, MS, Std
50	Hexyl hexanoate	Grass, Fruity	NBF	1602	1601	1	2	1	2	RI, Aroma, MS, Std
51	Ethyl 2-furoate	Paint, Pungent	NBF	1611	1608	1	1.5	-	1	RI, Aroma, MS, Std
52	Butyric acid	Cheese, Sweat	NBF/AF	1625	1615	4	3.7	4.3	3.7	RI, Aroma, MS, Std
53	Ethyl caprate	Fruity	NBF	1638	1636	2.5	2	1.5	1.5	RI, Aroma, MS, Std
54	Phenylacetaldehyde	Flower	NBF	1640	1641	2	2	3	2	RI, Aroma, MS, Std
55	Ethyl benzoate	Fruity	NBF	1658	1665	2.7	1.5	1	1	RI, Aroma, MS, Std
56	Isovaleric acid	Sweat, Sour	AF	1665	1663	3	3	2	2.5	RI, Aroma, MS, Std
57	Diethyl succinate	Fruity	NBF	1677	1668	2.5	2	2	1.3	RI, Aroma, MS, Std
58	n-Heptyl hexanoate	Fruity, Green	NBF	1693	1678	-	-	-	0.3	RI, Aroma, MS, Std
59	(2,2-diethoxyethyl) benzene	Medicine	NBF	1701	1710	2	1.3	3	2	RI, Aroma, MS, Std
60	Valeric acid	Sweat	NBF/AF	1733	1728	2	3	2.8	3.5	RI, Aroma, MS, Std
61	Ethyl phenylacetate	Flower, Sweet	NBF	1783	1773	-	1	1.5	1	RI, Aroma, MS, Std
62	4-Methylvaleric acid	Cheese	AF	1800	1795	1.5	1	1	1	RI, Aroma, MS, Std
63	Hexyl octanoate	Vegetables, Fruity	NBF	1796	1803	1	1.5	-	-	RI, Aroma, MS
64	Ethyl laurate	Fruity	NBF	1842	1838	1.5	1	-	-	RI, Aroma, MS, Std
65	Hexanoic acid	Sweat	AF	1846	1838	3	2.5	2	3	RI, Aroma, MS, Std
66	Phenethyl alcohol	Sweet	NBF	1906	1912	2.5	3	2.7	2	RI, Aroma, MS, Std
67	Heptanoic acid	Sweat	AF	1950	1946	1.7	1	2.5	0.5	RI, Aroma, MS, Std
68	Ethyl myristate	Flower	NBF	2049	2046	1.5	1	0.5	0.5	RI, Aroma, MS, Std
69	Octanoic acid	Sweat, Cheese	AF	2060	2055	2	2.5	1.5	1	RI, Aroma, MS, Std
70	p-Cresol	Pungent, Smoked	NBF	2076	2078	1.8	2	2	2	RI, Aroma, MS, Std
71	Ethyl cinnamate	Fruity, Honey	NBF	1893	2105	2	2.5	1.5	1	RI, Aroma, MS, Std
72	Ethyl oleate	Flower, Fruity	NBF	2476	2482	0.5	1	-	-	RI, Aroma, MS, Std

**Table 4:** Qualitative and quantitative analysis of 70 volatile compounds in different strong-aroma baijiu (μg/L).

No.	Aroma Compounds	Concentration (μg/L Unless Indicated Otherwise)				Odor Threshold	OAV			
		GJ	TZL	WLC	WLY		GJ	TZL	WLC	WLY
1	Ethyl acetate ^a^	1272.37 ± 308.24	488.07 ± 45.51	1678.06 ± 306.28	1496.05 ± 6.98	32.6	39.03	14.97	51.47	45.89
2	Ethyl propionate ^a^	7.82 ± 0.05a	8.7 ± 0.27a	5.44 ± 1.1b	8.36 ± 0.11a	19	<1	<1	<1	<1
3	Ethyl isobutyrate ^a^	11.08 ± 0.15a	6.6 ± 0.22b	4.84 ± 1.16c	3.85 ± 0.11c	0.058	191.03	113.79	83.45	66.38
4	Isobutyl acetate	347.64 ± 4.25a	316.97 ± 17.72a	222.87 ± 68.75b	266.44 ± 9.63ab	8	43.46	39.62	27.86	33.31
5	Ethyl butyrate ^a^	321.25 ± 16.77bcd	273.07 ± 3.92cd	388.13 ± 23.39ab	312.57 ± 46.22bcd	0.0815	3941.74	3350.59	4762.31	3835.23
6	Ethyl 2-methylbutyrate	2592.9 ± 79.74a	1931.15 ± 67.71ab	2263.26 ± 586.33a	1437.27 ± 44.52b	18	144.05	107.29	125.74	79.85
7	Ethyl isovalerate ^a^	2.86 ± 0.08c	3.33 ± 0.17a	2.07 ± 0.14d	2.69 ± 0.13c	0.007	408.57	475.71	295.71	384.29
8	Isoamyl acetate	1171 ± 10.98a	1039.43 ± 64.19a	648.09 ± 235.55b	992.19 ± 19.79a	94	12.46	11.06	6.89	10.56
9	Ethyl valerate ^a^	53.38 ± 6.9cd	70.11 ± 17.43bcd	91.64 ± 7.17abc	163.19 ± 8.38a	0.027	1976.98	2596.5	3394.03	6044.19
10	Amyl acetate	43.78 ± 0.21b	59.75 ± 1.37ab	121.53 ± 58.88a	117.63 ± 2.6a	1	43.78	59.75	121.53	117.63
11	Methyl hexanoate	477.74 ± 13.26df	689.87 ± 97.21bcd	1021.23 ± 128.54a	894.23 ± 43.94b	-	-	-	-	-
12	Ethyl 4-methylpentanoate	ND	665.88 ± 81.21b	572.24 ± 50.62b	1088.77 ± 184.5a	389	<1	1.71	1.47	2.8
13	Ethyl Hexanoate ^a^	1739.44 ± 614.95	2491.48 ± 743.33	2370.65 ± 754.34	3315.69 ± 825.68	0.055	31,626.2	45,299.6	43,102.7	60,285.3
14	Isoamyl butyrate	336.4 ± 36.59b	513.71 ± 41.81a	185.37 ± 17.13c	491.64 ± 38.91a	20	16.82	25.69	9.27	24.58
15	Propyl caproate	915.64 ± 79.5de	4508.12 ± 136.16b	2495.48 ± 232.86bcd	12,970.05 ± 3269.35a	12,800	<1	<1	<1	1.01
16	Ethyl heptanoate ^a^	37.42 ± 3.42d	77.12 ± 1.76b	37.18 ± 2.88d	125.43 ± 14.25a	13.2	2.84	5.84	2.82	9.5
17	Ethyl lactate^a^	918.33 ± 44.95bcd	758.51 ± 78.45cd	1059.93 ± 196.11ab	733.38 ± 28.88bcd	128	7.17	5.93	8.28	5.73
18	Butyl hexanoate ^a^	1.77 ± 0.19c	10.81 ± 0.3b	3.83 ± 0.14c	15.75 ± 2.84a	5.25	<1	2.06	<1	3
19	Ethyl caprylate ^a^	49.71 ± 3.96b	65.89 ± 2.68a	23.47 ± 1.41cde	36.66 ± 6.32b	0.013	3823.64	5068.62	1805.01	2819.95
20	Isopentyl hexanoate	3369.04 ± 375.13bc	6067.52 ± 194.79a	1092.86 ± 138.51de	3496.14 ± 663.34b	1400	2.41	4.33	<1	2.5
21	Pentyl hexanoate	113.1 ± 15.39b	711.4 ± 29.1b	281.12 ± 30.56b	1439.29 ± 261.98a	-	-	-	-	-
22	Furfuryl acetate	75.94 ± 3.38b	133.6 ± 2.1a	127.45 ± 23.56a	119.01 ± 6.97a	-	-	-	-	-
23	Hexyl hexanoate ^a^	4.35 ± 0.62bc	11.36 ± 0.08a	2.67 ± 0.31bc	11.47 ± 1.22a	1.89	2.3	6.01	1.41	6.07
24	Ethyl 2-furoate	58.27 ± 2.49b	142.6 ± 8.64a	42.54 ± 15.84b	60.21 ± 8.84b	-	-	-	-	-
25	Ethyl caprate	1526.52 ± 225.05a	723.38 ± 3.94b	292.56 ± 44.47def	424.11 ± 30.17bcde	1120	1.36	<1	<1	<1
26	Ethyl benzoate	160.09 ± 8.88a	68.42 ± 4.24b	31.98 ± 3.39d	47.95 ± 0.04c	-	-	-	-	-
27	Diethyl succinate	1654.63 ± 17.26a	790.49 ± 34.71b	619.41 ± 53.37c	503.92 ± 13.23d	-	-	-	-	-
28	n-Heptyl hexanoate	29.34 ± 3.39de	58 ± 0.26ab	26.13 ± 3.37cde	66.72 ± 4.58a	-	-	-	-	-
29	Ethyl phenylacetate	399.89 ± 5.03de	561.56 ± 53.29cde	2146.83 ± 80.64ab	589.01 ± 47.94c	407	<1	1.38	5.27	1.45
30	Hexyl octanoate	38.87 ± 3.02ab	51.52 ± 2.48a	25.79 ± 4.42ab	28 ± 3.09bc	-	-	-	-	-
31	Ethyl laurate	253.63 ± 8.05a	90.13 ± 10.38b	10.64 ± 1.6c	3.66 ± 1.04c	-	-	-	-	-
32	Ethyl myristate	517.76 ± 65.07a	147.35 ± 24.54de	141.29 ± 12.56def	38.59 ± 5.22fg	-	-	-	-	-
33	Ethyl cinnamate	985.17 ± 12.86b	1335.85 ± 24.14a	537.5 ± 40.55cd	223.91 ± 37.23f	-	-	-	-	-
34	Ethyl oleate	1250.76 ± 24.85a	637.06 ± 38.56bc	ND	434.29 ± 114.96cd	-	-	-	-	-
35	2-Butanol ^a^	18.31 ± 0.08d	39.23 ± 1.47b	23.96 ± 3.94c	53.57 ± 1.14a	50	<1	<1	<1	1.07
36	1-Propanol ^a^	144.93 ± 40.5bc	120.47 ± 35.54bc	151.75 ± 14.25abc	193.75 ± 48.97ab	54	2.68	2.23	2.81	3.59
37	2-Methyl-1-propanol ^a^	89.83 ± 9.24bc	182.19 ± 12.26a	68.54 ± 2.75de	80.14 ± 4.44cd	40	2.25	4.55	1.71	2
38	2-Pentanol ^a^	6.28 ± 0.14c	10.7 ± 0.61b	5.63 ± 1.3c	14.9 ± 0.49a	290	<1	<1	<1	<1
39	1-Butanol ^a^	160.33 ± 10.56ab	166.97 ± 10.56a	145.01 ± 10.31ab	40.03 ± 4.96e	2.73	58.73	61.16	53.12	14.66
40	3-Methyl-1-butanol ^a^	422.26 ± 6.66	234.54 ± 100.74	226.53 ± 25.8	248.12 ± 52.39	179	2.36	1.31	1.3	1.4
41	2-Heptanol	643.67 ± 265.02c	995.01 ± 115.11b	587.36 ± 116.91c	2228.27 ± 59.69a	1430	<1	<1	<1	1.56
42	1-Hexanol ^a^	23.34 ± 0.43h	62.67 ± 2.42a	55.74 ± 3.15b	62.17 ± 3.07a	5.37	4.35	11.67	10.4	11.6
43	Phenethyl alcohol ^a^	12.6 ± 1.09	11.64 ± 0.61	11.18 ± 0.97	12.95 ± 1.76	-	-	-	-	-
44	Acetaldehyde	2113.83 ± 31.87a	1947.04 ± 63.06a	1520.9 ± 303.93b	908.73 ± 21.59c	500	4.23	3.89	3.04	1.82
45	Isobutyraldehyde	1392.54 ± 12.92a	1478.29 ± 61.21a	1541.32 ± 133.85a	996.52 ± 111.37b	1300	1.07	1.14	1.19	<1
46	Hexanal	1261.48 ± 30.13b	1491.95 ± 134.41b	1550.84 ± 404.94b	2473.19 ± 67.3a	25.5	49.47	58.51	60.82	96.99
47	(E)-2-Octenal	39.06 ± 1.3	50.17 ± 0.63	45.14 ± 4.92	48.96 ± 12.3	15	2.60	3.34	3.01	3.26
48	Furfural ^a^	3.76 ± 0.08f	7.13 ± 0.51bcd	8.85 ± 0.7a	6.83 ± 0.54abc	0.122	30.78	58.48	72.6	56
49	Benzaldehyde	560.94 ± 20.33c	1110.31 ± 31b	1499.65 ± 340.25a	826.27 ± 45.47bc	-	-	-	-	-
50	(E)-2-Nonenal	54.01 ± 7.53	69.03 ± 6.47	36.78 ± 4.86	46.68 ± 6.49	51	1.06	1.35	<1	<1
51	5-Methyl furfural	69.39 ± 2.89b	120.16 ± 8.33a	142.42 ± 16.77a	138.37 ± 12.25a	-	-	-	-	-
52	Phenylacetaldehyde	528.46 ± 4.48f	858.96 ± 57.77d	1460 ± 107.04c	653.94 ± 90.33df	262	2.02	3.28	5.6	2.5
53	Acetic acid ^a^	503.39 ± 81.8ab	492.65 ± 33.97ab	567.04 ± 49.42a	555.77 ± 6.85a	160	3.15	3.08	3.54	3.47
54	Isobutyric acid ^a^	15.9 ± 2.28a	12.86 ± 1.41b	9.36 ± 1.2c	7 ± 0.25c	1.58	10.06	8.14	5.92	4.43
55	Butyric acid ^a^	137.58 ± 20.43abcd	98.74 ± 1.63de	164.47 ± 19.53abc	110.39 ± 15.69cd	0.964	142.71	102.43	170.61	114.51
56	Isovaleric acid ^a^	27.92 ± 3.55a	30.84 ± 2.81a	19.64 ± 1.96b	17.99 ± 0.76b	1.045	26.72	29.51	18.79	17.22
57	Valeric acid ^a^	2.94 ± 0.83f	17.86 ± 1.8cde	25.03 ± 11.53bcd	43.79 ± 1.47a	0.389	7.55	45.92	64.36	112.56
58	4-Methylvaleric acid	1114.36 ± 94.19a	877.03 ± 77.48b	773.49 ± 81.53bc	700.39 ± 25.05c	144	7.74	6.09	5.37	4.86
59	Hexanoic acid ^a^	381.07 ± 99.94	331.05 ± 34.65	347.34 ± 22.81	312.27 ± 28.62	2.52	151.22	131.37	137.83	123.92
60	Heptanoic acid ^a^	10.54 ± 1.67cd	7.58 ± 0.06ef	20.79 ± 1.82b	1.46 ± 0.05g	13.8	<1	<1	1.51	<1
61	Octanoic acid ^a^	22.62 ± 5.25b	29.76 ± 9.52a	15.59 ± 2.36cd	9.42 ± 0.74de	2.7	8.38	11.02	5.78	3.49
62	2,3-Butanedione ^a^	2.38 ± 0.38b	2.03 ± 0.91b	2.36 ± 0.15b	12.61 ± 2.87a	0.1	23.80	20.30	23.60	126.10
63	2-Pentanone ^a^	3.06 ± 0.04c	4.9 ± 0.17b	5.58 ± 1.22b	11.47 ± 0.39a	0.00138	2217.39	3550.72	4043.48	8311.59
64	2-Heptanone	194.22 ± 21.5	159.47 ± 2.51	350.66 ± 51.31	273.68 ± 14.84	140	1.39	1.14	2.50	1.95
65	Acetoin	206.03 ± 23.07b	494.6 ± 42.37b	4949.79 ± 157.23a	606.33 ± 34.89b	259	<1	1.91	19.11	2.34
66	Ethyl furfuryl ether ^a^	1.48 ± 0.15c	1.17 ± 0.06c	2.24 ± 0.17b	7.26 ± 0.57a	-	-	-	-	-
67	2-Acetylfuran	17.47 ± 2.33c	58.79 ± 1.42b	79.15 ± 16.63a	65.5 ± 3.22ab	-	-	-	-	-
68	Dimethyl trisulfide	63.2 ± 1.38ab	69.74 ± 1.05a	43.46 ± 8.63c	57.83 ± 2.53b	-	-	-	-	-
69	*p*-Cresol	2.01 ± 0.11c	4.79 ± 0.5a	3.68 ± 0.48b	3.73 ± 0.37b	0.054	37.22	88.70	68.15	69.07
70	(2,2-Diethoxyethyl)benzene	12.38 ± 1.58e	28.44 ± 3.24cd	37.23 ± 3.27c	36.11 ± 4.31c	-	-	-	-	-

Note: ND, not detected, and the content of compounds labeled with different letters has significant difference (*p* < 0.05). ^a^ The concentration of compound and its threshold is expressed in mg/L.

**Table 5:** Omission test of non-volatiles from strong-aroma baijiu model.

Number (a)	Category	Missing Compound	Triangle Test Significance (b)	Intensity (c)	Influence on Aroma Characteristics (d)
OM1	Organic acids (38)	Lactic acid, Glycolic acid, 2-Hydroxybutyric acid, Levulinic acid, 2-Furoic acid, Hydracrylic acid, 2-Hydroxy-2-methylbutyric acid, 3-Hydroxyisovaleric acid, 2-Hydroxyisocaproic acid, Benzoic acid, Benzeneacetic acid, (Z)-2-Butenedioic acid, Butanedioic acid, Methylsuccinic acid, (E)-2-Butenedioic acid, Benzenepropanoic acid, Mandelic acid, Malic acid, Hexanedioic acid, 3-Phenyllactic acid, 2-Phenyllactic acid, 4-Hydroxybenzoic acid, Dodecanoic acid, Tartaric acid, Suberic acid, Vanillic acid, Azelaic acid, Myristic acid, Sebacic acid, Pentadecanoic acid, Palmitelaidic acid, Palmitic acid, Heptadecanoic acid, (Z,Z)-9,12-Octadecadienoic acid, (Z)-Oleic acid, Stearic acid, Arachidic acid, Behenic acid	***	2.1	The sour and fruit aromas were significantly reduced, and the alcohol flavor was slightly enhanced
OM2	Carbohydrates (5)	α-D-Xylopyranose, D-Fructose, D-Glucose, D-Trehalose, Sucrose	ns		
OM3	Sugar alcohols (6)	Erythritol, Xylitol, Arabinitol, Ribitol, D-Mannitol, D-Glucitol	ns		
OM4	Alcohols (5)	2,3-Butanediol, Phenethylamine, Glycerol, Myo-Inositol, 2-Phenyl-1,2-propanediol	ns		
OM5	Monobasic acids (28)	Lactic acid, Glycolic acid, 2-Hydroxybutyric acid, Levulinic acid, 2-Furoic acid, Hydracrylic acid, 2-Hydroxy-2-methylbutyric acid, 3-Hydroxyisovaleric acid, 2-Hydroxyisocaproic acid, Benzoic acid, Benzeneacetic acid, Benzenepropanoic acid, Mandelic acid, 3-Phenyllactic acid, 2-Phenyllactic acid, 4-Hydroxybenzoic acid, Dodecanoic acid, Vanillic acid, Myristic acid, Pentadecanoic acid, Palmitelaidic acid, Palmitic acid, Heptadecanoic acid, (Z,Z)-9,12-Octadecadienoic acid, (Z)-Oleic acid, Stearic acid, Arachidic acid, Behenic acid	***	1.9	Sour and fruity aromas were significantly reduced
OM6	Dibasic acids (10)	2-Butenedioic acid, Butanedioic acid, Methylsuccinic acid, (E)-2-Butenedioic acid, Malic acid, Hexanedioic acid, Tartaric acid, Suberic acid, Azelaic acid, Sebacic acid	ns		

a. The aroma omission experiments were conducted by comparing the complete model with models where a specific class of compounds (OM 1–6) was omitted. b. Significance levels are indicated as follows: *** *p* < 0.001. c. During the test, participants were instructed to numerically rate the differences (0 = invalid, 1 = slightly different, 2 = quite different, 3 = completely different). d. The impact on aroma characteristics of the model when disregarding the specific mixture. ns represents no significant difference.

## Data Availability

The data presented in this study are available on request from the corresponding author. The data are not publicly available due to privacy or ethical restrictions.

## Supplementary Materials

The following supporting information can be downloaded at: https://www.mdpi.com/article/10.3390/foods14142490/s1, Table S1. Quantitative information of 72 volatile compounds in different strong aroma Baijiu (μg/L); Table S2. Qualitative and quantitative of 59 non-volatile compounds in different strong aroma Baijiu (μg/L).

## Abbreviations

The following abbreviations are used in this manuscript:

GC-MS/O	Gas chromatography-mass spectrometry/olfactometry
GC-TOF-MS	Gas chromatography-time of flight-mass spectrometry
BSTFA	Bis(trimethylsilyl)trifluoroacetamide
RI	Retention index
SPME	Solid-phase microextraction
IS	Internal standard
ANOVA	Analysis of variance
PCA	Principal component analysis
PLS-DA	Partial least squares discriminant analysis
OAV	Odor activity value
QDA	Quantitative descriptive analysis
