# Supplementary material for: Influences of Non-Volatile Components on the Aroma of Strong-Aroma Baijiu by Gas Chromatography-Olfactometry and Recombination-Omission Test
Source: Foods. 2025 Jul 16;14(14):2490. doi: 10.3390/foods14142490 (PMC12294591; doi:10.3390/foods14142490)
Supplement: Supplementary file 1 [file foods-14-02490-s001.zip › foods-3735847-supplementary.pdf]

Table S1 Quantitative information of 72 volatile compounds in different strong aroma Baijiu (μg/L)

NO.	Aroma compounds	RI	CAS	Standard curve					
		DB-WAX		Slope	Intercept	R^2	LOD (μg/L)	LOQ (μg/L)	Recovery
1	Ethyl acetate[a]	888	141-78-6	0.0746	-0.0014	0.9949	0.0039	0.0134	0.89
2	Ethyl propionate[a]	951	105-37-3	0.49	-0.00008	0.9998	0.0042	0.0137	1.01
3	Ethyl isobutyrate[a]	955	97-62-1	1.082	-0.0007	0.999	0.0049	0.0172	0.98
4	Isobutyl acetate	1018	110-19-0	1.508	0.0005	0.9975	0.0016	0.0051	0.94
5	Ethyl butyrate[a]	1035	105-54-4	0.109	-0.0111	0.9934	0.0030	0.0126	0.86
6	Ethyl 2-methylbutyrate	1050	7452-79-1	1.9755	0.0007	0.995	0.0027	0.0092	0.92
7	Ethyl isovalerate[a]	1068	108-64-5	0.1937	-0.0005	0.9968	0.0025	0.0104	1.05
8	Isoamyl acetate	1124	123-92-2	2.8331	0.0008	0.9965	0.0024	0.0069	0.91
9	Ethyl valerate[a]	1134	539-82-2	0.1438	0.0014	0.9986	0.0021	0.0067	0.97
10	Amyl acetate	1175	628-63-7	5.9402	-0.001	0.9965	0.0009	0.0034	0.92
11	Methyl hexanoate	1184	106-70-7	0.3547	0.0004	0.9959	0.0020	0.0065	0.95
12	Ethyl 4-methylpentanoate	1190	25415-67-2	0.1738	0.0015	0.9948	0.0012	0.0045	1.05
13	Ethyl Hexanoate[a]	1272	142-92-7	0.0445	0.0012	0.9976	0.0008	0.0029	0.90
14	Isoamyl butyrate	1259	106-27-4	1.3163	-0.00004	0.9985	0.0002	0.0010	0.98
15	Caproic acid propyl ester	1316	626-77-7	0.9006	0.0013	0.9934	0.0093	0.0392	0.95
16	Ethyl heptanoate[a]	1331	106-30-9	0.5657	0.0111	0.9922	0.0083	0.0362	0.89
17	Ethyl lactate[a]	1347	97-64-3	0.123	-0.0055	0.9969	0.0084	0.0312	0.93
18	Butyl hexanoate[a]	1407	626-82-4	1.2792	0.0042	0.9972	0.0010	0.0039	0.96
19	Ethyl caprylate[a]	1435	106-32-1	1.3018	0.016	0.9954	0.0018	0.0069	0.94
20	Isopentyl hexanoate	1451	2198-61-0	3.2079	0.0043	0.9966	0.0087	0.0355	0.94
21	Pentyl hexanoate	1501	540-07-8	2.1927	0.0002	0.9992	0.0066	0.0295	0.99

22	Furfuryl acetate	1539	623-17-6	0.5371	0.0014	0.9977	0.0012	0.0064	0.96
23	Hexyl hexanoate[a]	1602	6378-65-0	2.2169	-0.0031	0.9959	0.0010	0.0036	0.93
24	Ethyl 2-furoate	1611	614-99-3	3.732	0.0009	0.9987	0.0002	0.0009	0.96
25	Ethyl caprate	1638	110-38-3	4.2	-0.0018	0.9991	0.0093	0.0324	0.96
26	Ethyl benzoate	1658	93-89-0	2.7176	0.0006	0.9997	0.0120	0.0830	0.99
27	Diethyl succinate	1677	123-25-1	0.7125	-0.0009	0.9978	0.0034	0.0089	1.05
28	n-Heptyl hexanoate	1693	6976-72-3	3.2808	-0.0002	0.9943	0.0253	0.0801	0.89
29	Ethyl phenylacetate	1783	101-97-3	2.5894	0.0009	0.9992	0.0043	0.0275	1.02
30	hexyl ester Octanoic acid	1796	1117-55-1	5.9267	-0.0009	0.9934	0.0175	0.0476	0.94
31	Ethyl laurate	1842	106-33-2	2.5062	0.0021	0.9924	0.0032	0.0124	0.92
32	Ethyl myristate	2049	124-06-1	1.4172	0.0005	0.9974	0.0015	0.0096	0.99
33	Ethyl cinnamate	1893	103-36-6	1.9632	0.0002	0.9999	0.0134	0.0824	1.01
34	Ethyl oleate	2476	6114-18-7	0.0173	-0.000003	0.9941	0.1562	0.5748	0.91
35	2-Butanol[a]	1024	78-92-2	0.0371	-0.0000009	0.9997	0.0621	0.2145	1.01
36	1-Propanol[a]	1036	71-23-8	0.2391	0.0033	0.999	0.0302	0.1046	0.96
37	2-Methyl-1-propanol[a]	1092	78-83-1	0.4851	-0.0124	0.9916	0.0313	0.1113	0.90
38	2-Pentanol[a]	1118	6032-29-7	0.1934	0.00008	0.9997	0.0026	0.0089	0.98
39	1-Butanol[a]	1142	71-36-3	0.1171	0.0024	0.9997	0.0405	0.1312	1.01
40	3-Methyl-1-butanol[a]	1209	123-51-3	0.2672	-0.0008	0.9968	0.0156	0.0505	0.92
41	2-Heptanol	1322	543-49-7	3.3355	-0.0006	0.9987	0.0015	0.0048	1.03
42	1-Hexanol[a]	1355	111-27-3	0.0664	0.0002	0.9994	0.0843	0.3021	1.03
43	Phenethyl alcohol[a]	1906	60-12-8	0.2275	-0.0238	0.9967	0.0839	0.3042	0.93
44	Acetaldehyde	714	75-07-0	0.3428	0.0007	0.9967	0.0008	0.0031	0.95
45	Isobutyraldehyde	821	78-84-2	0.2948	0.0001	0.9993	0.0011	0.0039	0.99
46	Hexanal	1078	66-25-1	0.645	-0.0001	0.9996	0.0021	0.0066	0.97

47	*(E)*-2-Octenal	1430	2548-87-0	6.9781	-0.0009	0.9992	0.0022	0.0071	0.98
48	Furfural[a]	1462	98-01-1	0.041	0.0011	0.9988	0.0628	0.2045	0.93
49	Benzaldehyde	1520	100-52-7	3.095	0.0019	0.994	0.0003	0.0014	0.93
50	*(E)*-2-Nonenal	1534	18829-56-6	0.5677	-0.00001	0.9999	0.0045	0.0249	0.98
51	5-Methyl furfural	1570	620-02-0	0.5192	0.0002	0.9982	0.0044	0.0145	0.97
52	Phenylacetaldehyde	1640	122-78-1	0.0914	0.0001	0.9927	0.0677	0.2838	0.85
53	Acetic acid[a]	1449	64-19-7	0.1581	0.0014	0.9927	0.0063	0.0230	0.86
54	Isobutyric acid[a]	1563	79-31-2	0.4838	-0.0012	0.9932	0.0378	0.0987	0.91
55	Butyric Acid[a]	1625	107-92-6	0.0413	0.0007	0.9924	0.0714	0.2642	0.88
56	Isovaleric acid[a]	1665	503-74-2	0.5026	-0.0004	0.998	0.0487	0.1789	1.03
57	Valeric acid[a]	1733	109-52-4	0.0318	0.0017	0.9966	0.0653	0.2344	0.90
58	4-Methylvaleric acid	1800	646-07-1	1.1878	-0.007	0.9934	0.0531	0.1487	1.09
59	Hexanoic acid[a]	1846	142-62-1	0.0112	0.0049	0.9997	0.0603	0.2373	0.97
60	Heptanoic acid[a]	1950	111-14-8	0.1077	0.0055	0.9975	0.06847	0.2674	0.94
61	Octanoic acid[a]	2060	124-07-2	0.2025	0.0143	0.9965	0.0643	0.2478	0.93
62	2,3-Butanedione[a]	975	431-03-8	0.0436	-0.0003	0.999	0.0031	0.0104	0.97
63	2-Pentanone[a]	1020	107-87-9	0.3761	-0.00006	0.9997	0.0025	0.0082	1.00
64	2-Heptanone	1182	110-43-0	1.5876	-0.0012	0.9968	0.0004	0.0011	0.94
65	Acetoin	1287	513-86-0	1.1965	-0.0023	0.998	0.0024	0.0073	0.95
66	2,6-diethyl-Pyrazine	1444	13067-27-1	-	-	-	-	-	-
67	Tetramethyl pyrazine	1469	1124-11-4	-	-	-	-	-	-
68	Ethyl furfuryl ether[a]	1291	6270-56-0	1.9907	0.0016	0.9979	0.0016	0.0057	0.95
69	2-Acetylfuran	1490	1192-62-7	0.6834	0.0006	0.9923	0.0024	0.0073	1.08
70	Dimethyl trisulfide	1377	3658-80-8	7.2822	-0.0012	0.9978	0.0018	0.0062	1.02
71	p-Cresol	2076	106-44-5	0.4205	-0.0006	0.9938	0.0019	0.0065	0.91

72	(2,2-Diethoxyethyl)benzene	1701	6314-97-2	1.7594	-0.00004	0.9967	0.2600	0.9801	0.98

Table S2 Qualitative and quantitative of 59 non-volatile compounds in different strong aroma Baijiu (μg/L)

NO.	Aroma compounds	RI	CAS	Standard curve						Concentrations (μg/L)			
		HP-5		Slope	Intercept	R^2	LOD (μg/L)	LOQ (μg/L)	Recovery rate	GJ	TZL	WLC	WLY
						Acids							
1	Lactic acid, 2TMS derivative[a]	1066	17596-96-2	0.2392	2.1479	0.9979	9.72	30.43	96%	1359.46 ±76.83ab	1040.92 ±257.68c	1508.54 ±188.03a	504.32± 43.64d
2	Glycolic acid, 2TMS derivative	1081	33581-77-0	1.0488	0.0649	0.9992	1.21	5.34	99%	800.37± 64.1d	1435.35 ±160.62b	316.41± 61.57e	420.97± 15.16e
3	2-Hydroxybutyric acid, 2TMS derivative	1136	55133-93-2	1.4464	0.0028	0.994	0.98	4.49	90%	409.2± 22.3a	321.4± 22.8b	151.6± 27.3e	121.7± 4.2f
4	Levulinic acid, TMS derivative	1125	55557-12-5	0.2052	0.029	0.998	0.36	1.17	101%	213.4± 40.9cd	498.65± 44.95a	200.45± 46.45d	193± 15.6d
5	2-Furoic acid, TMS derivative	1131	55887-53-1	0.6406	-0.0165	0.9929	2.12	5.37	87%	520.85± 31.25c	1170.25 ±71.25a	337.15± 58.85d	875.45± 21.85b
6	Hydracrylic acid, 2TMS derivative	1151	55162-32-8	-	-	-	-	-	-	439.13± 38.99cde	669.04± 61.43ab	564.46± 121.76bc	367.64± 15.18e
7	3-Hydroxybutyric acid, 2TMS derivative	1167	55133-94-3	1.4036	0.0017	0.9934	0.48	3.17	90%	218.47± 1.65fg	425.11± 39.99b	289.44± 75.32de	168.51± 18.14g

8	2-Hydroxy-2-methylbutyric acid, 2TMS derivative	1170	55557-18-1	0.7957	0.0158	0.9929	0.78	2.52	86%	149.5±3.4d	548.55±35.05a	148.1±34.1d	215.8±5.2c
9	3-Hydroxyisovaleric acid, 2TMS derivative	1216	55124-90-8	0.6184	-0.0102	0.997	1.79	5.25	97%	231.05±3.95b	323±25.1a	106.05±20.65e	131.7±11.3d
10	2-Hydroxyisocaproic acid, 2TMS derivative	1248	54890-08-3	0.9524	0.0031	0.9965	2.28	7.71	106%	9769.1±2474.62a	4350.95±302.41b	3419.25±173.64bcd	1338.47±65.41e
11	Benzoic acid, TMS derivative	1249	2078-12-8	1.7147	0.0429	0.9966	0.92	2.76	105%	352.95±34.25e	685.52±37.57cd	1415.06±351.89a	493.66±7.5de
12	Benzeneacetic acid, TMS derivative	1302	2078-18-4	1.6933	0.0501	0.9978	0.89	3.12	97%	576.04±63.76de	1166.33±76.02b	1118.1±269.19b	855.54±27.05c
13	*(Z)*-2-Butenedioic acid, 2TMS derivative	1312	23508-82-9	0.9825	0.0022	0.9985	0.21	0.75	98%	292.2±42.7b	351.9±24.1a	91.3±17.2e	238±17.6c
14	Butanedioic acid, 2TMS derivative	1321	40309-57-7	1.2965	0.0044	0.9993	0.24	1.14	101%	1273.7±75a	536.45±53.05c	134.85±29.45fg	148.0±20.05f
15	Methylsuccinic acid, 2TMS derivative	1331	55557-26-1	0.9941	0.0101	0.9988	1.39	4.81	96%	139.4±1.9b	191.7±16.4a	81.05±10.95d	6.2±0.5f
16	Glyceric acid, 3TMS derivative	1344	38191-87-6	-	-	-	-	-	-	114.72±5.97b	181.45±12.54a	44.23±11.37de	57±4.4d
17	*(E)*-2-Butenedioic acid, 2TMS derivative	1353	17962-03-7	1.6499	0.0042	0.9989	0.34	1.74	93%	20.7±0.5c	41.55±1.45a	14.9±4.5d	27.4±2b
18	Benzenepropanoic acid, TMS derivative	1414	21273-15-4	0.7993	0.0015	0.9983	1.58	4.72	95%	1641±104.19a	1321.06±92.75b	451.23±108.58de	314.03±19.57fg
19	Mandelic acid, 2TMS derivative	1491	2078-19-5	1.8303	-0.0191	0.9959	0.48	1.69	92%	18.85±2.15c	25.5±0.2ab	25.5±3.7ab	23.4±2.2b

20	Malic acid, 3TMS derivative	1497	38166-11-9	0.2564	-0.0046	0.9957	0.31	1.56	93%	582.15± 12.75c	1274.5± 96.2a	190.5± 26.2f	329.55± 40.15e
21	Hexanedioic acid, 2TMS derivative	1514	18105-31-2	0.3382	0.0102	0.9995	2.13	7.34	102%	27.15± 7.25b	0.25± 0.25c	27.7± 23.8b	13.75± 3.25bc
22	3-Phenyllactic acid, 2TMS derivative	1599	27750-45-4	1.1618	-0.0022	0.9979	1.72	5.38	104%	627.4± 57.4b	334.15± 22.55c	68.4± 14.2e	48.45± 2.95e
23	2-Phenyllactic acid, 2TMS derivative	-	82326-12-3	1.1996	0.0067	0.9906	1.39	4.52	113%	599.71± 55.69b	316.17± 22.06c	58.27± 13.71e	38.98± 2.71e
24	4-Hydroxybenzoic acid, 2TMS derivative	1635	2078-13-9	1.3352	0.0099	0.9988	2.37	7.51	95%	7.27± 1.18cd	7.54± 0.82c	5.83± 1.43cd	4.37± 0.51d
25	Dodecanoic acid, TMS derivative	1655	55520-95-1	0.1043	-0.0137	0.9959	2.59	6.36	93%	1068.78 ±74.19a	534.08± 7.93b	212.23± 22.95de	352.52± 2.03c
26	Tartaric acid, 4TMS derivative	1665	18602-86-3	0.0972	-0.0003	0.9971	0.27	0.85	105%	621.6± 76.9bc	678.75± 92.55b	601.1± 59.7bc	526.3± 14.5cd
27	Suberic acid, 2TMS derivative	1707	43199-48-0	0.2605	0.0051	0.9984	2.29	7.48	103%	59.85± 3.35c	20.45± 2.05de	76.5± 11.1b	19.9± 0.2e
28	Vanillic acid, 2TMS derivative	1776	2078-15-1	0.8728	-0.0042	0.9994	1.87	6.38	97%	11.35± 0.05cd	14.5± 1.1bc	10.2± 1.4d	9.65± 0.35d
29	Azelaic acid, 2TMS derivative	1806	17906-08-0	0.2926	-0.0002	0.9934	0.54	2.38	91%	115.2± 0.9bc	107.95± 16.45bcd	120.85± 35.95b	50.6± 11.3e
30	Myristic acid, TMS derivative	1850	18603-17-3	0.2067	-0.0603	0.9994	0.95	3.46	101%	2104.16 ±0.09a	1027.93 ±31.95c	574.33± 59.83d	873.73± 15.62c
31	Sebacic acid, 2TMS derivative	1904	18408-42-9	0.4062	-0.0023	0.995	0.58	2.31	93%	20± 0.8bcd	19.2± 1.9bcd	25± 6.5b	14.4± 1.6d
32	Pentadecanoic acid,	1950	74367-22-9	0.1629	-0.0166	0.9994	0.79	2.38	100%	391.69±	231.54±	179.36±	233.51±

	TMS derivative									4.31a	11.05bc	18.83cd	3.49bc
33	Palmitelaidic acid, TMS derivative	-	82326-15-6	0.1581	-0.0396	0.9992	3.39	11.72	92%	996.15±60.95a	780.87±22.11b	817.92±127.67b	1032.83±1.06a
34	Palmitic acid, TMS derivative[a]	2050	55520-89-3	0.2652	-0.0002	0.9981	0.26	1.15	90%	14.54±0.48a	8.31±0.56c	7.74±0.92cd	11.91±0.46b
35	Heptadecanoic acid, TMS derivative	2146	55517-58-3	0.2092	-0.0284	0.9997	1.16	3.79	101%	293.02±13.66a	256.13±7.39abc	237.24±26.74c	298.12±3.73a
36	*(Z,Z)*-9,12-Octadecadienoic acid， TMS derivative	2212	56259-07-5	0.0335	-0.0047	0.9985	0.75	2.38	98%	14140.02±137.78a	5268.56±161.84d	7245.85±818.79b	3848.32±355.34e
37	*(Z)*-Oleic acid, TMS derivative[a]	2218	21556-26-3	0.1022	-0.014	0.9994	1.47	5.34	99%	2.85±0.1cd	4.52±0.45b	3.28±0.87c	6.62±0.36a
38	Stearic acid, TMS derivative	2246	18748-91-9	0.2957	-0.0619	0.9989	0.34	1.16	102%	2263.7±241.25bc	1999.12±224.71bcd	1874.08±351.45cd	3071.33±239.72a
39	Arachidic acid, TMS derivative	2447	55530-70-6	0.0557	-0.0147	0.9998	0.59	2.19	100%	1105.91±89.69ab	976±117.53bc	800.5±118.55cd	1293.13±152.24a
40	Behenic acid, TMS derivative	2644	74367-36-5	0.0918	-0.0445	0.9999	1.48	6.07	101%	1112.82±55.87a	985.64±82.97abc	904.61±93.24bc	1149.56±89.84a
41	Lignoceric acid, TMS derivative	2838	74367-37-6	-	-	-	-	-	-	10.33±0.19a	7.58±1.11b	5.8±1.58b	10.63±1.84a
						Alcohols							
42	2,3-Butanediol, 2TMS derivative[a]	1032	53274-85-4	0.5743	0.1023	0.9997	15.26	55.29	97%	10.05±1.4d	13.78±1.34bc	19.28±1.89a	11.15±2.41cd

43	Phenethylamine, TMS derivative[a]	1224	10433-33-7	0.2275	-0.0238	0.9967	8.39	30.42	93%	11.18± 0.97	12.95± 1.76	12.6± 1.09	11.64± 0.61
44	Glycerol, 3TMS derivative	1289	6787-10-6	2.7013	-0.0129	0.9973	1.27	4.39	94%	674.98± 57.29b	656.06± 17.76b	725.09± 38.62b	592.83± 15.89b
45	2-Phenyl-1,2-propanediol, 2TMS derivative	-	294847-15-7	-	-	-	-	-	-	537.43± 12.9a	334.36± 4.74b	147.65± 31.94de	87.75± 3.99f
46	Myo-Inositol, 6TMS derivative	2129	2582-79-8	1.3633	0.0027	0.9974	0.94	3.45	94%	5.96± 0.32bc	9.77± 1.09b	6± 1.97bc	0.28± 0.01c
	Sugar alcohols												
47	Erythritol, 4TMS derivative	1508	25258-02-0	1.1865	0.0086	0.9968	0.56	1.78	92%	8.75± 0.68b	44.17± 4.32b	17.25± 5.68b	34.46± 1.01b
48	Xylitol, 5TMS derivative	1741	-	1.0243	-0.0024	0.999	0.64	3.38	104%	12.66± 0.64b	20± 1.94b	21± 3.92b	11.02± 0.35b
49	Arabinitol, 5TMS derivative	1737	14199-73-6	1.2581	-0.0124	0.9988	0.44	2.46	105%	20± 0.58b	26.05± 1.56b	26.04± 3.78b	17.19± 1.89b
50	Ribitol, 5TMS derivative	1747	32381-53-6	0.8928	-0.0038	0.9996	0.35	1.29	98%	27.36± 0.81b	23.47± 1.58b	21.77± 3.78b	8.48± 0.99b
51	D-Mannitol, 6TMS derivative	1964	14317-07-8	1.2263	-0.0165	0.9981	0.77	2.56	96%	17.63± 0.28c	32.21± 1.45b	19.26± 0.75c	17.39± 1.17c
52	D-Glucitol, 6TMS derivative	1980	14199-80-5	0.6578	-0.0121	0.9993	0.39	1.67	99%	21.27± 0.27bc	24.52± 0.04b	21.45± 0.43bc	19.88± 0.04c
	Carbohydrates												
53	α-D-Xylopyranose, 4TMS derivative	1762	14251-20-8	1.4364	-0.2504	0.9921	0.38	1.5	93%	190.44± 0.38b	195.05± 0.6b	189.51± 1.42b	191.52± 2.38b
54	D-Fructose, 5TMS derivative	1842	19126-98-8	1.186	0.0572	0.9936	0.67	2.88	87%	24.09± 0.35cd	560.98± 28.75a	15.03± 2.54cd	9.07± 0.47d

55	D-Glucose, 5TMS derivative	1926	6736-97-6	3.1568	0.3608	0.9998	0.79	2.62	89%	109.78±22.69d	1120.19±50.58a	115.21±24.57d	72.63±2.23de
56	D-Trehalose, 7TMS derivative	-	60065-05-6	1.7846	-0.0187	0.9968	0.13	0.45	91%	43.51±2.15a	37.51±1.38b	31.36±1.32c	18.94±0.29d
57	Sucrose, 8TMS derivative	2712	19159-25-2	1.3136	0.0385	0.9963	0.43	1.58	88%	ND	278.93±23.19a	ND	ND
						Amino acids							
58	L-5-Oxoproline, 2TMS derivative	1522	30274-77-2	1.0637	-0.0067	0.9979	0.05	0.48	102%	55.91±6.33b	70.72±12.19b	41.19±4.44b	22.13±3.5b
						Esters							
59	monoethyl ester-Succinic acid, (TMS)	1251	959313-61-2	0.5635	0.0066	0.9983	1.23	4.62	98%	1580.48±121.32a	704.56±43.79b	240.3±61.36de	173.78±34.47e

Note: ND means not detected, and the content of compounds labeled with different letters (a, b, c, d, e, f, g, h, i) has significant difference (P<0.05). The average content of compounds represented by letter a is the highest, and the average content of compounds represented by letter i is the lowest. [a] means the concentration of compound is expressed in mg/L.
